# Impact of COVID-19 Infection on Cardiorespiratory Fitness, Sleep, and Psychology of Endurance Athletes—CAESAR Study

**DOI:** 10.3390/jcm12083002

**Published:** 2023-04-20

**Authors:** Daniel Śliż, Szczepan Wiecha, Jakub S. Gąsior, Przemysław Seweryn Kasiak, Katarzyna Ulaszewska, Marcin Lewandowski, Marcin Barylski, Artur Mamcarz

**Affiliations:** 1 3rd Department of Internal Medicine and Cardiology, Medical University of Warsaw, 04-749 Warsaw, Poland; 2 School of Public Health, Postgraduate Medical Education Center, 01-813 Warsaw, Poland; 3 Department of Physical Education and Health, Faculty in Biala Podlaska, Jozef Pilsudski University of Physical Education in Warsaw, 21-500 Biala Podlaska, Poland; 4 Department of Pediatric Cardiology and General Pediatrics, Medical University of Warsaw, 02-091 Warsaw, Poland; 5 Students’ Scientific Group of Lifestyle Medicine, 3rd Department of Internal Medicine and Cardiology, Medical University of Warsaw, 04-749 Warsaw, Poland; 6 Department of Pharmacology and Clinical Pharmacology Collegium Medicum, Cardinal Stefan Wyszyński University in Warsaw, 00-927 Warsaw, Poland; 7 Department of Internal Medicine and Cardiac Rehabilitation, Medical University of Lodz, 90-419 Łódź, Poland

**Keywords:** COVID-19, endurance athletes, mental health, sleep, cardiopulmonary exercise testing, cardiorespiratory fitness, exercise capacity, physical exercise, psychology

## Abstract

COVID-19 has a deteriorating impact on health which is especially important for endurance athletes (EAs) who need to maintain continuity of training. The illness affects sleep and psychology, which influence sport performance. The aims of this study were: (1) to assess the consequences of mild COVID-19 on sleep and psychology and (2) to assess the consequences of mild COVID-19 on cardiopulmonary exercise test (CPET) results. A total of 49 EAs (males = 43, 87.76%; females = 6, 12.24%; age = 39.9 ± 7.8 years; height = 178.4 ± 6.8 cm; weight = 76.3 ± 10.4 kg; BMI = 24.0 ± 2.6 kg·m^−2^) underwent a maximal cycling or running CPET pre- and post-COVID-19 and completed an original survey. Exercise performance deteriorated after COVID-19 (maximal oxygen uptake, VO_2max_ = 47.81 ± 7.81 vs. 44.97 ± 7.00 mL·kg·min^−1^ pre- and post-infection, respectively; *p* < 0.001). Waking up at night affected the heart rate (HR) at the respiratory compensation point (RCP) (*p* = 0.028). Sleep time influenced pulmonary ventilation (*p* = 0.013), breathing frequency (*p* = 0.010), and blood lactate concentration (Lac) (*p* = 0.013) at the RCP. The maximal power/speed (*p* = 0.046) and HR (*p* = 0.070) were linked to the quality of sleep. Stress management and relaxation techniques were linked with VO_2max_ (*p* = 0.046), maximal power/speed (*p* = 0.033), and maximal Lac (*p* = 0.045). Cardiorespiratory fitness deteriorated after mild COVID-19 and was correlated with sleep and psychological indices. Medical professionals should encourage EAs to maintain proper mental health and sleep after COVID-19 infection to facilitate recovery.

## 1. Introduction

The ongoing Coronavirus disease 2019 (COVID-19) pandemic has had a significant impact on various aspects of people’s lives for almost three years [1] Not only does the disease itself pose a threat to individuals’ physical health, but misinformation and a lack of trust in medical treatment and preventative methods can also contribute to increased uncertainty and stress [2,3,4]. COVID-19 infection has been shown to harm the heart function of patients who survived the infection [5], especially in people who have a problem with their weight [6,7]. A greater tendency toward a reduced left ventricular ejection fraction, end-diastolic volume, and stroke volume was found, which can negatively affect the physical activity of patients [8]. Clinicians and psychologists are therefore searching for new coping strategies to address the mental health impacts of the pandemic [9]. The lockdown measures implemented to slow the spread of the virus have also greatly affected people’s lifestyles, activities, and mental health [4]. It is also well-known that COVID-19 disease and lockdown can also affect the sleeping patterns and endurance of patients; for this reason, mental health, sleep, and endurance are crucial concepts affected by COVID-19 [10].

The COVID-19 pandemic is a particularly difficult time in the lives of EAs and other people. EAs from Poland, Romania, and Slovakia had the highest level of mental stress during the fourth wave [11]. EAs also adopted different coping strategies that affected mental health differently [12]. Returning to regular training and physical fitness may not be easy due to mental aspects [13]. The proper maintenance of activity can prevent further stress related to the pandemic and lockdown [14]. Physical activity is associated with the reduced hospitalization, intensive care unit admissions, and mortality of COVID-19 patients. People who mainly perform resistance and endurance exercises are less likely to be hospitalized [15]. Research results suggest that among athletes, women have a higher risk of developing post-COVID-19 symptoms and fatigue than men [16,17]. COVID-19 and the lockdown had an impact on adverse lifestyle changes and the sports results achieved by EA [18]. Coaches, medical doctors, and EAs attempt to counteract these problems to return to previous levels of competition and fitness [19]. Moreover, some may need rehabilitation [20]. Regarding mental health, 12 months after the disease, patients present symptoms of mental disorders and a lack of concentration and focus that increases with the severity of the infection [21]. Significant improvement was noted 2 years after infection, which is reassuring, but it should be noted that experiencing the consequences of COVID-19 infection on mental health can influence the future health state of an EA [22]. It was reported that the course of mental disorders related to COVID-19 depends on age and sex [23,24]. Commonly reported post-illness psychiatric symptoms are anxiety (6.5% to 63%), depression (4% to 31%), and post-traumatic stress disorder (12.1% to 46.9%). Patients reported a lower quality of life up to 3 months after illness [25]. These data show that this is not a problem for individuals; however, it can affect the everyday functioning and motivation of a large group of patients.

Another aspect is the impact of the disease on patients’ sleep. Patients often report insomnia related to infection; however, this is usually mild [26]. During the pandemic, endurance athletes were found to experience changes in their training, competition, and sleep patterns which can negatively affect their performance [27]. Results from the study indicate that athletes who reported sleep disturbances had a lower endurance performance, and average marathon finishing times decreased during the pandemic. A meta-analysis showed the post-COVID-19 neurological and neuropsychiatric changes: on average, 31% of patients experience sleep disorders [28]. This is a worrying phenomenon because sleep is a key aspect of the proper functioning of the body. This effect is especially challenging for EAs, as sleep loss is associated with poorer athletic performance as well as exercise efficiency [29].

The aim of our study is twofold: to understand how contracting COVID-19 affects sleep and mental health and to evaluate how a previous mild COVID-19 infection impacts the results of endurance performance scores among EAs. These are aspects that we will struggle with during the pandemic, and EAs will have to find solutions to counteract them until a fully effective vaccine or drug is found and the population shows a greater willingness to use vaccinations [30].

## 2. Materials and Methods

### 2.1. General Study Information

We conducted a study that included a double CPET assessment and a mental health and sleep questionnaire. EAs underwent the CPET assessment before and after a COVID-19 infection. During the second CPET assessment, they also received the survey. Participants were recruited, and selected EAs were invited for the post-infection exercise tests in the period between June 2021 and December 2022. All pre- and post-infection CPETs were performed under controlled protocol at a single laboratory, the SportsLab sports diagnostics center (SportsLab, Warsaw, Poland). Participants underwent CPET before and after the disease with the same exercise modality (cycling or running). The interval between infection and both CPETs were measured to control the effect of time elapsed. The sample consisted of amateur EAs at various levels of fitness according to reference standards for VO_2max_ [31,32]. After infection and directly before the second CPET, each EA underwent a medical evaluation by a physician (a cardiology or internal medicine specialist), which consisted of taking their medical history, a physical examination, a 12-lead ECG, echocardiography, and a complete blood count. The EAs were screened for ongoing long-lasting COVID-19 (e.g., respiratory and circulatory) consequences preventing them from performing CPET.

Inclusion criteria: (1) an interval between the first CPET and COVID-19 infection <3 years, (2) a mild COVID-19 infection (which did not require hospitalization) confirmed by PCR or antigen test, (3) participation in the survey, (4) no ongoing, long-lasting COVID-19 consequences preventing the EA from engaging in the CPET (e.g., related to circulatory and respiratory systems), and (5) presenting a negative COVID-19 PCR or antigen test.

Exclusion criteria: (1) EAs with a pulmonary condition (COPD, uncontrolled bronchial asthma, and blood saturation <95%), (2) EAs with a cardiovascular disease (arrhythmia confirmed by ECG, ischemia of myocardium, QT prolongation confirmed by ECG, morphological heart abnormalities confirmed by echocardiography, and uncontrolled hypertension > 160/100 mmHg) [33], (3) EAs with a present mental condition, (4) EAs with an orthopedic condition that prevented them from performing the CPET, (5) deviations in CBC (leukocytosis > 10,000·mm^−3^, anemia with blood hemoglobin <10 g·dL^−1^).

**Figure 1:**
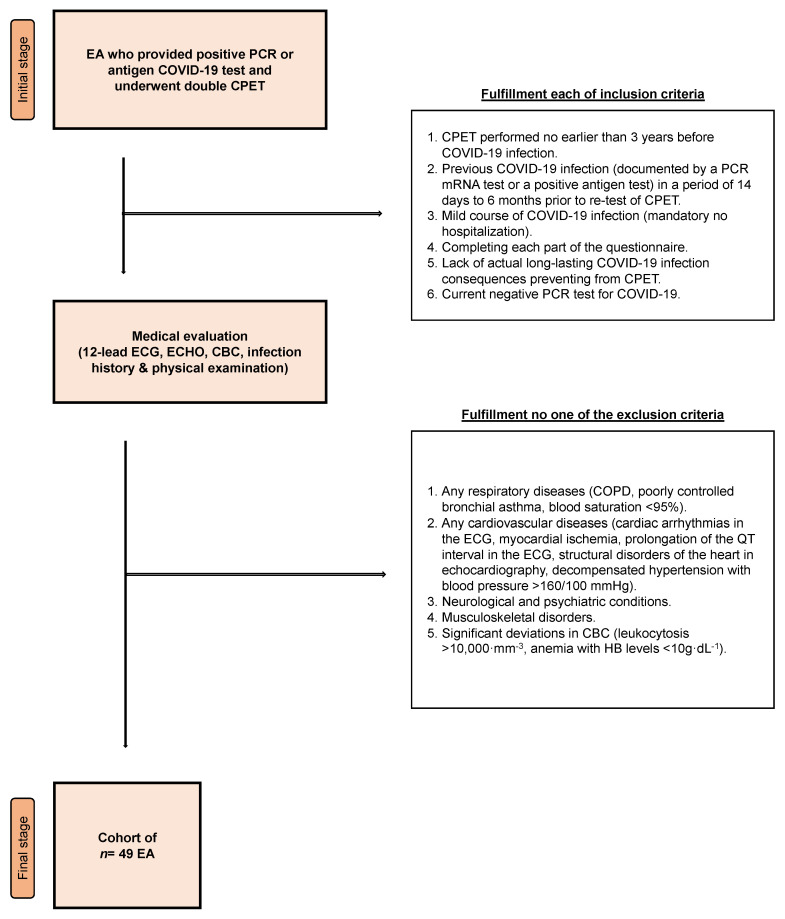
Recruitment procedure. Abbreviations: EA—endurance athlete; PCR—polymerase chain reaction; COVID-19—Coronavirus disease 2019; CPET—cardiopulmonary exercise test; ECG—12-lead electrocardiogram; ECHO—echocardiography examination; CBC—complete blood count; COPD—chronic obstructive pulmonary disease; HB, blood hemoglobin.

A visual representation of the recruitment procedure is provided in Figure 1.

### 2.2. Cohort Description

Among the 49 EAs recruited for this study, 87.8% (n = 43) were males and 12.2% (n = 6) were females. The males were 40.7 (7.0) years old and 178.5 (6.8) cm in height, while the females were 38.1 (6.4) years old and 178.4 (6.9) cm in height. There were 63.3% (n = 31) running and 26.7% (n = 18) cycling exercise examinations. The participants had to be prespecified in running or cycling, but could also add other supplemental trained disciplines. Of the participants, 30.6% (n = 15) declared additional disciplines which included triathlon, football, and martial arts. Of the cohort, 8.2% (n = 4) trained for 1–2 years, 28.6% (n = 14) for 3–5 years, 38.8% (n = 19) for 6–10 years, and 21.3% (n = 12) had >10 years of training experience. Of the EAs, 46.9% (n = 23) withdrew from some type of competition due to experiencing a COVID-19 infection. Individuals assessed their general health status on the −5/0/+5 scale as 4.8 (0.5) pre- and as 4.1 (0.5) post-COVID-19 infection, while 20.4% (n = 10) of them declared suffering from COVID-19 consequences lasting longer than 2 weeks in the past. The time from the first to the second CPET was 591.7 (282.2) days. The period between the pre-COVID-19 CPET and the termination of the infection (defined as negative PCR) was 436.4 (290.4) days, while the period between the post-COVID-19 CPET and the termination of the infection was 155.3 (82.52) days.

### 2.3. Questionnaire

We used the previously validated PaLS (Pandemic against LifeStyle) questionnaire [34], which covered the following domains: (1) basic information about the subjects, their training experience, health status, and infection details (20 questions), (2) mental health, coping strategies, and mood state (14 questions) and (3) sleep habits (13 questions). The basic information section consisted of questions examining demographic data, primary sports discipline, training and competition experience, and any previous, long-lasting COVID-19 consequences. We added a point to each section in which the EAs rated on a scale of −5/0/+5 the impact of the COVID-19 pandemic, the restrictions introduced, the course of the disease, and the resulting lifestyle changes. Negative values represented a harmful effect, positive values represented a positive effect, and 0 meant no association. The scale allowed for adjustments to noticed changes in intensities.

#### 2.3.1. Mental Health Section

The EAs’ mental health was assessed by the original questions. The EAs were asked about coping strategies (rapid return to work, neglect or acceptance of the current condition, usage of stimulants and alcohol, seeking support among others, expanding knowledge about the virus, joking about the infection, and usage of relaxation techniques), infection-related mood changes (concentrating on the situation, more often suffering negative emotions) and observed mental health disorders (giving up, a more positive or more negative outlook on life, criticizing themselves, and strong expressions of negative emotions). EAs could choose the following answers: (1) “I did not try this method”, (2) “I used it in my everyday life, but only to a small extent”, (3) “I used this method often, or it was one of the basic methods to cope with COVID-19 induced stress”, and (4) “I used this method regularly”.

#### 2.3.2. Sleep Section

Sleep was assessed using the Athens Insomnia Scale [35], and the three additional questions related to usual habits: hour of going into bed (participants declared the precise time at which they went to bed), sleep time (described in hours and minutes), and time spent in front of devices emitting blue light (also described in hours and minutes).

### 2.4. CPET Procedure and Somatic Measurements

Each subject performed an intensity-adjusted, maximal-effort–limited CPET with either running (mechanical treadmill, h/p/Cosmos quasar, Nussdorf–Traunstein, Germany) or cycling (cycle ergometer, RBM elektronik-automation GmbH, Leipzig, Germany). The selected modality was the same post-infection as pre-infection. During the pre-infection period, CPET participants chose their modality based on their preference and primary sport discipline. During examinations, constant beath-by-breath gas exchange (Hans Rudolph V2 Mask, Hans Rudolph Inc, Shawnee, KS, USA), blood lactate (Super GL2 analyzer, Müller Gerätebau GmbH, Freital, Germany), and cardiopulmonary (Cosmed Quark CPET device, Rome, Italy) monitoring were used. The cycling test began with 3–5 min of freewheel pedaling, followed by a gradual increase in intensity (20 Watts/2 min for females and 30 Watts/2 min for males). The running protocol also began with a 3–5-minute warmup at a speed varying between 7–12 km per hour and a constant 1% inclination, followed by a gradual increase in speed (1 km/2 min both for females and males). The CPET was terminated when the subject declared volitional exhaustion, and maximal effort was additionally confirmed by a heart rate (HR) or maximal oxygen uptake (VO_2max_) plateau (lack of growth in exercise parameter with growing CPET resistance). Participants were verbally encouraged by the physiologist to achieve a maximum score. The anaerobic threshold (AT) and respiratory compensation point (RCP) were determined based on actually recommended guidelines [36]. Before each exercise test, a body composition examination was performed (the Tanita body analyzer, Tanita, MC 718, Tokyo, Japan). The used multifrequency was 5 kHz/50 kHz/250 kHz. Obtained endpoints were weight, height, body mass index (BMI), lean mass, body fat percentage (BF), fat mass, VO_2_, HR, pulmonary ventilation (VE), speed (for running CPET), power (for cycling CPET), breathing frequency (f_R_), and blood lactate concentration (Lac).

### 2.5. Data Analysis

The results are shown as the number (n) and percentage (%) for categorical variables and as the average with standard deviation for continuous variables. Data are shown in line with the APA Guidelines (https://apastyle.apa.org/; accessed on 16 March 2023). The Shapiro–Wilk test was used to evaluate the normal distribution. Relationships between CPET and somatic measures (weight, BMI, lean mass, BF, fat mass, VO_2_, HR, VE, running speed, cycling power, f_R_, and Lac) and questionnaire results (sleep and mental health outcomes) were assessed via the Kruskal–Wallis rank ANOVA. Differences between pre-/post-COVID-19 results for exercise and somatic performance (weight, BMI, lean mass, BF, fat mass, VO_2_, HR, VE, running speed, cycling power, f_R_, and Lac) were obtained using Student’s *t*-test for independent means. The sample size was calculated with the use of the G∗Power software (version 3.1.9.2; Düsseldorf, Germany). The total necessary number of participants reached its minimal effective value. A value of *p* = 0.05 was considered a significance borderline. Data analysis was performed in STATISTICA (version 13.3, StatSoft Polska Sp. z o.o., Kraków, Poland) and SPSS (version 28; IBM SPSS, Chicago, IL, USA).

## 3. Results

### 3.1. CPET Performance

The CPET performance, stratified by infection status, is presented in Table 1. Between pre- and post-exercise tests, significant differences were found in key parameters, such as VO_2_ at the AT (35.0 (6.4) vs. 32.4 (5.9) ml·kg·min^−1^, *p* < 0.001), RCP (43.9 (7.3) vs. 40.5 (6.6) ml·kg·min^−1^, *p* < 0.001), and maximal (47.8 ((7.8) vs. 45.0 (7.0) ml·kg·min^−1^, *p* < 0.001). VO_2_ was higher before infection. We also observed a deterioration in HR at the AT (145.1 (10.8) vs. 141.1 (10.0) bpm, *p* < 0.001) and RCP (168.8 (9.0) vs. 165.1 (9.7) bpm, *p* < 0.001). Other significantly different variables were running speed at the AT (*p* = 0.044) and the RCP (*p* < 0.001), VE at the RCP (*p* < 0.001), and Lac at the RCP (*p* = 0.013).

### 3.2. Sleep and Mental Health

A description of participants’ responses with the mean range, where applicable, is presented in Table 2 for sleep and Table 3 for mental health. We are presenting only significant (*p* < 0.05) results owing to a large amount of possible response–CPET variable combinations. Briefly, the mental health of our EAs showed a strong link to their CPET performance. Awakenings during the night influenced HR at the RCP (H(2) = 7.2; *p* = 0.028). One EA who described it as a considerable problem also noticed the highest HR at the RCP (mean range = 99.9 vs. 71.4 vs. 55.1). The sufficient total sleep duration was linked with the highest VE at the RCP when compared to slightly and markedly insufficient total sleep duration (H(2) = 8.7; *p* = 0.013; mean range = 30.4 vs. 18.5 vs. 29.7). Similar associations were observed for f_R_ at the RCP (H(2) = 4.5; *p* = 0.104) and Lac at the RCP (H(2) = 8.7; *p* = 0.013). Sleep quality correlated with maximal power or speed, both relative and absolute VO_2_ at the RCP and maximal, VE at the RCP and maximal, and maximal HR (each *p* < 0.05). All precise results stratified by answer type and exercise variable have been shown in Table 4, part A.

Interestingly, we found a much less significant relationship between self-reported mental health and sports performance. Briefly, our EAs applied different coping strategies, and their habits to improve their mental state varied significantly. Undertaking activities to improve one’s situation (e.g., by learning more about COVID-19) was linked to a lean body mass (H(3) = 8.2; *p* = 0.042). It is worth noting that joking about the COVID-19 infection influenced up to five CPET variables: HR at the AT (H(3) = 8.2; *p* = 0.042), absolute VO_2_ at the RCP (H(3) = 9.1; *p* = 0.029), VE at the RCP (H(3) = 8.3; *p* = 0.041), maximal relative VO_2_ (H(3) = 8.0; *p* = 0.047) and maximal absolute VO_2_ (H(3) = 10.6; *p* = 0.014). The expression of negative emotions (e.g., by shouting loudly or arguing with others) correlated with VE at the AT (H(3) = 10.9; *p* = 0.012), and EAs who often used this method observed the highest VE values (mean range = 44.0 vs. 42.0 vs. 34.4 and 20.4). The use of relaxation techniques altered running speed or cycling power (H(2) = 6.8; *p* = 0.033), maximal relative VO_2_ (H(2) = 6.1; *p* = 0.046), and maximal Lac (H(2) = 6.2; *p* = 0.045). We did not observe any other significant association between declared mental health state or habit and CPET performance. All Kruskal–Wallis H test scores from the mental health section are presented in Table 4, part B.

## 4. Discussion

In our study, we showed the impact of having mild COVID-19 on EAs’ mental health and sleep, as well as their correlation with CPET scores. The main findings were: (1) episodes of awakening during sleep affected HR at the RCP, (2) sufficient total sleep duration compared to slightly and markedly insufficient total sleep duration was linked with the highest VE at the RCP, (3) the quality of sleep correlated with maximal power or speed and maximal HR, (4) EAs adopted different strategies of coping with stress, which was associated with the influence on lean body mass, and (5) CPET parameters were influenced by EAs’ individual behaviors and habits. This article focuses on the outcomes of mild COVID-19 infection on sleep and mental health. Other possibly affecting covariables (including the participants’ sex, age, CPET modality, nutrition, training regimen, and previous sports experience) were analyzed in the remaining CAESAR manuscripts [37,38].

The effect of sleep deprivation and sleep duration on athletic performance, reaction time, accuracy, strength, and endurance in EAs has been proven in many studies [39]. In our study, EAs who reported insufficient sleep time had significant changes in parameters such as pulmonary ventilation, breathing frequency, and blood lactate concentration at the respiratory compensation point; lactate changes are mainly influenced by sleep deprivation at the end of the night [40]. In contrast, increasing sleep time or introducing naps could improve reaction time, alertness, vigor and mood, as well as prevent fatigue [41]. The reduction in endurance parameters may be partially linked to poor sleep hygiene; therefore, it is important to practice good sleep hygiene. For EAs, the correct quality and duration of sleep is essential because it affects physical and mental regeneration, which are necessary for achieving high sports results [42]. The reduction in endurance parameters may be partially linked to poor sleep hygiene; thus, it is important to maintain it. Among young EAs, up to 41% do not comply with the rules of sleep hygiene [43]. They exhibit behaviors such as exposure to blue light before falling asleep, extended wake-up time, and eating meals before falling asleep. Delayed onset and awakening after falling asleep and the presence of sleep phases unaffected by varying training severity suggest a questionable recovery in athletes after intense training [44]. The quality of sleep among EAs surveyed in our study changed parameters such as speed, power, oxygen uptake, absolute oxygen uptake, pulmonary ventilations at the respiratory compensation point and maximal oxygen uptake, absolute maximal oxygen uptake, maximal heart rate, and maximal pulmonary ventilation compared to the results before they became ill with with COVID-19.

COVID-19 also affected the mental health of endurance athletes: 22.2% of EAs reported mood deterioration or symptoms of depression during the COVID-19 pandemic. Comparably, only 3.8% reported such signs when asked about the pre-pandemic period [45]. This is an alarming result, considering that mental health affects CPET scores in EAs. Moreover, as the pandemic continues, this condition is becoming worse. In a comparison of the results from 2020 to 2021, despite better access to possibilities of training, mental problems increased from 36% to 80% [46]. Among young EAs whose activity level decreased during the pandemic, an improvement in the quality of mental life was noticed after returning to regular activity [47]. Mood, stress levels, and overall mental health among EAs may be lower even at 8 weeks after COVID-19 infection [48], which consequently adversely affects athletic performance and attitude to training. It is worth underlining that people who have recovered from COVID-19 are at risk of memory loss, anxiety, depression, and even post-traumatic stress disorder (PTSD) compared to individuals who have not had the disease [49]. This paper was directed at the impact of the disease on asymptomatic and mildly symptomatic EAs. As stated by Petek et al., this was the most common course of COVID-19 infection in the athletic population [50]. Even non-hospitalized EAs are more likely to develop anxiety, trauma- and stress-related disorders, or fatigue [51]. Augustin et al., found that up to 14% of patients report fatigue at a 7-month follow up, and females are considered a higher-risk group [52]. EAs incorporate various treatment strategies. Briefly, progressive muscle relaxation techniques have a positive impact on anxiety and sleep quality during the ongoing COVID-19 disease [53]. Thus, it is worth considering as a strategy for EAs during and after the disease. It is important to provide EAs with comprehensive care and assistance in recovering from COVID-19 and in later returning to sports competition under stressful circumstances [54,55].

Declared negative emotions were correlated with VE at the AT. This result may be influenced by the fact that stressful circumstances can enhance the response of EAs to exertion and mobilize them to higher performance [56]. The impact of the relaxation techniques on the Lac among EAs was confirmed by a study conducted on runners [57]. After six months, athletes using meditation or autogenic training had significantly reduced Lac after exercise compared to the control group. Changes in VO_2max_ were not significant, however. Findings provided by Solberg et al., may be extrapolated to the post-pandemic period because mindfulness techniques could improve performance, endurance, and cognitive functions [58]. Finally, we found that joking about the disease affected HR, VO_2,_ and VE; however, the basic mechanism remains unclear. We underline that the primary goal of this paper is not to investigate the causative mechanisms but to draw attention to the links between CPET changes, well-being, and experiencing a COVID-19 infection in the athletic population. Thus, we recommend further studies to examine the physiologic reasons for the above-described results.

### Limitations

The survey results were based on self-reported answers and may not accurately reflect participants’ actual habits. We did not collect data about the training periodization phases during the time of the study. The time gap between the pre- and post-infection CPET tests may have an impact on the results due to variations in fitness levels among participants. It is important to exercise caution when applying the findings to other situations and to conduct additional research to confirm the conclusions about the effects of mild COVID-19 infection on exercise performance, sleep, and mental health.

## 5. Conclusions

The quality of sleep and mental health is greatly impacted by both the ongoing pandemic and contracting COVID-19. It is essential for EAs to have access to professional medical and psychological support. Adopting effective coping strategies can aid in the treatment and prevention of mental health issues. There is also a connection between mental health and sleep habits and athletic performance. The course of COVID-19 infection and the lifestyle of athletes have an impact on cardiorespiratory capacity and CPET test results. Therefore, those working with EAs, such as coaches, clinicians, and psychologists, should be aware of the potential effects of mild COVID-19 infection and take steps to protect their health, including providing appropriate treatment recommendations.

## Figures and Tables

**Table 1:** CPET performance.

Variable	Pre-COVID-19	Post-COVID-19	*p*-Value
Weight (kg)	76.6 (10.0)	76.7 (10.9)	0.951
BMI (kg·m^−2^)	24.0 (2.5)	24.0 (2.7)	0.931
Lean mass (kg)	63.4 (7.6)	63.5 (8.0)	0.774
BF (%)	17.1 (4.7)	16.9 (5.1)	0.604
Fat mass (kg)	13.3 (4.7)	13.2 (5.2)	0.848
VO_2AT_ (ml·kg·min^−1^)	35.0 (6.5)	32.4 (6.0)	<0.001 *
VO_2ATa_ (ml·min^−1^)	2650.0 (470.9)	2446.1 (400.3)	<0.001 *
HR_AT_ (beats·min^−1^)	145.1 (10.9)	141.1 (10.1)	0.001 *
VE_AT_ (l·min^−1^)	70.8 (18.7)	68.1 (14.7)	0.090
S_AT_ (km·h^−1^)	11.4 (1.4)	11.1 (1.3)	0.044 *
P_AT_ (Watts)	162.8 (25.9)	154.8 (25.9)	0.066
f_RAT_ (breaths·min^−1^)	32.1 (9.0)	32.1 (8.1)	0.706
Lac_AT_ (mmol·L^−1^)	2.0 (0.9)	2.1 (0.9)	0.630
VO_2RCP_ (ml·kg·min^−1^)	43.9 (7.4)	40.5 (6.7)	<0.001 *
VO_2RCPa_ (ml·min^−1^)	3324.3 (512.9)	3063.7 (440.1)	<0.001 *
HR_RCP_ (beats·min^−1^)	168.8 (9.2)	165.1 (9.8)	<0.001 *
VE_RCP_ (l·min^−1^)	106.8 (21.7)	98.9 (18.3)	<0.001 *
S_RCP_ (km·h^−1^)	14.3 (1.9)	13.8 (1.5)	<0.001 *
P_RCP_ (Watts)	245.2 (42.0)	232.2 (39.7)	0.061
f_RRCP_ (breaths·min^−1^)	41.3 (8.7)	40.1 (8.9)	0.876
Lac_RCP_ (mmol·L^−1^)	4.9 (1.4)	4.3 (1.1)	0.013 *
VO_2max_ (ml·kg·min^−1^)	47.8 (8.0)	45.0 (7.1)	<0.001 *
VO_2maxa_ (ml·min^−1^)	3623.5 (552.1)	3406.0 (474.5)	<0.001 *
HR_max_ (beats·min^−1^)	180.8 (10.1)	179.8 (10.0)	0.273
VE_max_ (l·min^−1^)	143.0 (26.9)	138.50 (23.9)	0.068
S_max_ (km·h^−1^)	16.6 (1.6)	16.4 (1.7)	0.264
P_max_ (Watts)	310.0 (37.2)	312.2 (49.1)	0.811
f_Rmax_ (breaths·min^−1^)	58.9 (14.4)	57.3 (11.0)	0.959
Lac_max_ (mmol·L^−1^)	9.7 (2.3)	9.6 (2.4)	0.880

Abbreviations: COVID-19—Coronavirus disease 2019; BMI—body mass index; BF—body fat; VO_2AT_—oxygen uptake at the anaerobic threshold; VO_2ATa_—absolute oxygen uptake at the anaerobic threshold; HR_AT_—heart rate at the anaerobic threshold; VE_AT_—pulmonary ventilation at the anaerobic threshold; S_AT_—speed at the anaerobic threshold; P_AT_—power at the anaerobic threshold; f_RAT_—breathing frequency at the anaerobic threshold; Lac_AT_—blood lactate concentration at the anaerobic threshold; VO_2RCP_—oxygen uptake at the respiratory compensation point; VO_2RCPa_ absolute oxygen uptake at the respiratory compensation point; HR_RCP_—heart rate at the respiratory compensation point; VE_RCP_—pulmonary ventilation at the respiratory compensation point; S_RCP_—speed at the respiratory compensation point; P_RCP_—power at the respiratory compensation point; f_RRCP_—breathing frequency at the respiratory compensation point; Lac_RCP_—blood lactate concentration at the respiratory compensation point; VO_2max_—maximal oxygen uptake; VO_2maxa_—absolute maximal oxygen uptake; HR_max_—maximal heart rate; VE_max_—maximal pulmonary ventilation; S_max_—maximal speed—P_max_—maximal power; f_Rmax_—maximal breathing frequency; Lac_max_—maximal blood lactate concentration. Speed is presented for treadmill CPET (n = 29), and power is presented for a cycle ergometer CPET (n = 18). Significant values (*p* < 0.05) have been marked with an asterisk (*).

**Table 2:** Results of answers to questions related to sleep.

Question	Answer Type				Lack of Answer
	n (%)	n (%)	n (%)	n (%)	n (%)
Sleep induction	No problem	Slightly delayed	Markedly delayed	Very delayed or did not sleep at all	2 (4.1)
	22 (44,9)	16 (32.7)	7 (14.3)	2 (4.1)	
Awakenings during the night	No problem	Minor problem	Considerable problem	Serious problem or did not sleep at all	2 (4.1)
	8 (16.3) 71.4 for HR_RCP_	27 (55.1) 55.1 for HR_RCP_	12 (24.5) 99.9 for HR_RCP_	0 (0.0)	
Final awakening	Not earlier	A little earlier	Markedly earlier	Much earlier or did not sleep at all	3 (6.1)
	28 (57.1)	15 (30.6)	3 (6.1)	0 (0.0)	
Total sleep duration	Sufficient	Slightly insufficient	Markedly insufficient	Very insufficient or did not sleep at all	2 (4.1)
	19 (38.8) 30.4 for VE_RCP_ 29.2 for f_RRCP_ 24.9 for Lac_RCP_	25 (51.0) 18.5 for VE_RCP_ 20.4 for f_RRCP_ 13.9 for Lac_RCP_	3 (6.1) 29.7 for VE_RCP_ 21.3 for f_RRCP_ 18.0 for Lac_RCP_	0 (0.0)	
Sleep quality	Satisfactory	Slightly unsatisfactory	Markedly unsatisfactory	Very unsatisfactory or did not sleep at all	2 (4.1)
	22 (44.9) 27.2 for S_RCP_/P_RCP_ 29.4 for VO_2RCPa_ 29.2 for VO_2RCP_ 29.1 for VE_RCP_ 27.6 for VO_2max_ 27.5 for VO_2maxa_ 27.3 for HR_max_ 25.9 for VE_max_	22 (44.9) 19.3 for S_RCP_/P_RCP_ 17.6 for VO_2RCPa_ 18.0 for VO_2RCP_ 18.3 for VE_RCP_ 18.8 for VO_2max_ 18.6 for VO_2maxa_ 19.4 for HR_max_ 19.8 for VE_max_	3 (6.1) 35.3 for S_RCP_/P_RCP_ 31.3 for VO_2RCPa_ 28.7 for VO_2RCP_ 28.7 for VE_RCP_ 35.3 for VO_2maxa_ 38.0 for VO_2maxa_ 33.8 for HR_max_ 40.7 for VE_max_	0 (0.0)	
Well-being during the day	Normal	Slightly decreased	Markedly decreased	Very decreased	2 (4.1)
	28 (57.1) 27.8 for S_RCP_/P_RCP_ 28.3 for S_max_/P_max_	19 (38.8) 18.5 for S_RCP_/P_RCP_ 17.6 for S_max_/P_max_	0 (0.0)	0 (0.0)	
Functioning capacity during the day	Normal	Slightly decreased	Markedly decreased	Very decreased	3 (6.1)
	26 (53.1)	19 (38.8)	1 (2.0)	0 (0.0)	
Sleepiness during the day	None	Mild	Considerable	Intense	2 (4.1)
	5 (10.2)	28 (57.1)	13 (26.5)	1 (2.0)	
What hour did you usually get out of bed in the morning	7.1 (2.5)				4 (8.2)
Hours of actual sleep per night	6.8 (1.0)				
Hours spend in front of the screen of devices emitting blue light per day	7.0 (2.9)				3 (6.1)
Self-assessed impact of COVID-19 pandemic and imposed restrictions sleep (in -5/0/+5 scale)	0.4 (1.3)				2 (4.1)

Abbreviations: COVID-19—Coronavirus disease 2019; HR_RCP_—heart rate at the respiratory compensation point; VE_RCP_ pulmonary ventilation at the respiratory compensation point; f_RRCP_—breathing frequency at the respiratory compensation point; Lac_RCP_—blood lactate concentration at the respiratory compensation point; S_RCP_—speed at the respiratory compensation point; P_RCP_—power at the respiratory compensation point; VO_2RCP_—oxygen uptake at the respiratory compensation point; VO_2max_—maximal oxygen uptake; VO_2maxa_—absolute maximal oxygen uptake; HR_max_—maximal heart rate; VE_max_—maximal pulmonary ventilation; S_max_—maximal speed—P_max_—maximal power. Speed was considered for running CPET, while power was considered for cycling CPET. Data are shown as number (n) and (percentage) for categorical variables or as mean and (standard derivation). Kruskal–Wallis mean range was shown only in case of significant differences (*p* < 0.05). Where the CPET score significantly correlated (*p* < 0.05) with psychological or sleep indices, we added exact mean ranks calculated from the Kruskal–Wallis rank ANOVA. To clarify, the higher the mean rank value, the higher was the value achieved for the particular CPET parameter by the endurance athlete with the given answer.

**Table 3:** Results of answers to questions related to mental health.

Question	Answer Type				Lack of Answer
	I Did Not Try This Method	I Used It in My Everyday Life, but Only to a Small Extent	I Used This Method Often, or It Was One of the Basic Methods to Cope with COVID-19 Induced Stress	I Used This Method Regularly	
	n (%)	n (%)	n (%)	n (%)	n (%)
I wanted to return to work and duties as soon as possible to stop thinking about my illness	24 (49.0)	8 (16.3)	4 (8.2)	11 (22.4)	2 (4.1)
I was concentrating very hard on the situation I found myself in	27 (55.1)	11 (22.4)	0 (0.0)	8 (16.3)	3 (6.1)
I told myself „It can’t be true that I am infected with COVID-19”	43 (87.8)	4 (8.2)	0 (0.0)	1 (2.0)	1 (2.0)
I used alcohol or other stimulants to improve my mood	37 (75.5)	7 (14.3)	1 (2.0)	1 (2.0)	3 (6.1)
I gave up after trying to cope with the whole situation	42 (85.7)	1 (2.0)	3 (6.1)	0 (0.0)	3 (6.1)
I was looking for support from family, friends and other people	33 (67.3)	8 (16.3)	1 (2.0)	4 (8.2)	3 (6.1)
I undertook activities to improve my situation by expanding my knowledge about COVID-19	17 (34.7) 24.9 for lean mass	13 (26.5) 15.4 for lean mass	3 (6.1) 35.0 for lean mass	13 (26.5) 27.2 for lean mass	3 (6.1)
I was looking for positives in the situation I found myself in	12 (24.5)	7 (14.3)	8 (16.3)	19 (38.8)	3 (6.1)
I criticized myself for not taking precautions enough, which resulted in COVID-19 infection	40 (81.6)	6 (12.2)	0 (0.0)	0 (0.0)	3 (6.1)
I tried to joke about the COVID-19 infection	18 (36.7) 27.7 for HR_AT_ 25.6 for VO_2RCP_ 27.3 for VE_RCP_ 24.9 for VO_2max_ 26.3 for VO_2maxa_	11 (22.4) 23.5 for HR_AT_ 28.5 for VO_2RCP_ 22.5for VE_RCP_ 28.6 for VO_2max_ 28.0 for VO_2maxa_	4 (8.2) 41.5 for HR_AT_ 30.3 for VO_2RCP_ 34.3 for VE_RCP_ 30.3 for VO_2max_ 31.3 for VO_2maxa_	13 (26.5) 15.2 for HR_AT_ 14.3 for VO_2RCP_ 15.8 for VE_RCP_ 15.1 for VO_2max_ 13.5 for VO_2maxa_	3 (6.1)
I quickly accepted the state I was in	3 (6.1)	7 (14.3)	20 (40.8)	16 (32.7)	3 (6.1)
I expressed my negative emotions expressively by screaming loudly or arguing with others	37 (75.5) 20.4 for VE_AT_	7 (14.3) 34.4 for VE_AT_	1 (2.0) 44.0 for VE_AT_	1 (2.0) 42.0 for VE_AT_	3 (6.1)
I tried to improve my mood through religious practices or meditation relaxation techniques	35 (71.4) 22.3 for S_max_/P_max_ 23.0 for VO_2max_ 13.7 for Lac_max_	6 (12.2) 35.8 for S_max_/P_max_ 34.2 for VO_2max_ 21.7 for Lac_max_	0 (0.0)	5 (10.2) 17.2 for S_max_/P_max_ 14.4 for VO_2max_ 7.8 for Lac_max_	3 (6.1)
Self-assessed impact of COVID-19 pandemic and imposed restrictions mental health (in -5/0/+5 scale)	0.6 (1.8)				2 (4.1)

Abbreviations: COVID-19—coronavirus disease 2019; HR_AT_—heart rate at the anaerobic threshold; VO_2RCP_—oxygen uptake at the respiratory compensation point; VE_RCP_ pulmonary ventilation at the respiratory compensation point; VO_2max_—maximal oxygen uptake; VO_2maxa_—absolute maximal oxygen uptake; VE_AT_—pulmonary ventilation at the anaerobic threshold; S_max_—maximal speed—P_max_—maximal power; Lac_max_—maximal blood lactate concentration. Speed was considered for running CPET, while power was considered for cycling CPET. Data are shown as number (n) and (percentage) for categorical variables or as mean and (standard derivation). Kruskal–Wallis mean range was shown only in case of significant differences (*p* < 0.05). Where the CPET score significantly correlated (*p* < 0.05) with psychological or sleep indices, we added exact mean ranks calculated from the Kruskal–Wallis rank ANOVA. To clarify, the higher the mean rank value, the higher was the value achieved for the particular CPET parameter by the endurance athlete with the given answer.

**Table 4:** Relationships between sleep, mental health, and CPET performance.

CPET Variable	Survey Question	*p*-Value
Part A. Sleep		
HR_RCP_	Awakenings during the night	0.028 *
VE_RCP_	Total sleep duration	0.013 *
f_RRCP_	Total sleep duration	0.010 *
Lac_RCP_	Total sleep duration	0.013 *
S_RCP_/P_RCP_	Sleep quality	0.046 *
VO_2RCPa_	Sleep quality	0.011 *
VO_2RCP_	Sleep quality	0.018 *
VE_RCP_	Sleep quality	0.027 *
VO_2max_	Sleep quality	0.034 *
VO_2maxa_	Sleep quality	0.019 *
HR_max_	Sleep quality	0.070
VE_max_	Sleep quality	0.032 *
S_RCP_/P_RCP_	Well-being during the day	0.023 *
S_max_/P_max_	Well-being during the day	0.007 *
Part B. Mental health		
Lean mass	Undertaking activities to improve one’s situation	0.042 *
HR_AT_	Joking about the COVID-19 infection	0.042 *
VO_2RCP_	Joking about the COVID-19 infection	0.029 *
VE_RCP_	Joking about the COVID-19 infection	0.041 *
VO_2max_	Joking about the COVID-19 infection	0.047 *
VO_2maxa_	Joking about the COVID-19 infection	0.014 *
VE_AT_	Expressing negative emotions expressively	0.012 *
S_max_/P_max_	Improving mood through religious practices or meditation relaxation techniques	0.033 *
VO_2max_	Improving mood through religious practices or meditation relaxation techniques	0.046 *
Lac_max_	Improving mood through religious practices or meditation relaxation techniques	0.045 *

Abbreviations: CPET—cardiopulmonary exercise test; HR_RCP_—heart rate at the respiratory compensation point; VE_RCP_ pulmonary ventilation at the respiratory compensation point; f_RRCP_—breathing frequency at the respiratory compensation point; Lac_RCP_—blood lactate concentration at the respiratory compensation point; S_RCP_—speed at the respiratory compensation point; P_RCP_—power at the respiratory compensation point; VO_2RCPa_ absolute oxygen uptake at the respiratory compensation point; VO_2RCP_—oxygen uptake at the respiratory compensation point; VO_2max_—maximal oxygen uptake; VO_2maxa_—absolute maximal oxygen uptake; HR_max_—maximal heart rate; VE_max_—maximal pulmonary ventilation; S_max_—maximal speed—P_max_—maximal power; HR_AT_—heart rate at the anaerobic threshold; VE_AT_—pulmonary ventilation at the anaerobic threshold; Lac_max_—maximal blood lactate concentration. Owing to a large number of combinations between the survey question and CPET variable, only significant results (with *p* < 0.05) were presented. *p*-values were calculated using the Kruskal–Wallis H test. Speed was considered for running CPET, while power was considered for cycling CPET. Significant values (*p* < 0.05) are marked with an asterisk (*).

## Data Availability

The data presented in this study are available upon request from the corresponding author. The data are not publicly available due to not obtaining consent from respondents to publish the data.
